# P-2196. Utilizing Telemedicine to Engage Rural Patients with Substance Use Disorders in Low Barrier Hepatitis C Treatment

**DOI:** 10.1093/ofid/ofae631.2350

**Published:** 2025-01-29

**Authors:** Kristi C Hill, Sherilyn Brinkley, Maria C Latimer, Patricia Tichnell, Tracy Agee, Jackie Bittner, Mark S Sulkowski, Oluwaseun Falade-Nwulia

**Affiliations:** Johns Hopkins University, Baltimore, Maryland; Johns Hopkins University, Baltimore, Maryland; Johns Hopkins University School of Medicine, Baltimore, Maryland; Johns Hopkins, Baltimore, Maryland; Johns Hopkins University, Baltimore, Maryland; Johns Hopkins, Baltimore, Maryland; Johns Hopkins University School of Medicine, Baltimore, Maryland; Johns Hopkins University, Baltimore, Maryland

## Abstract

**Background:**

Many rural areas have high hepatitis C virus (HCV) prevalence driven by substance use disorders (SUD) but few treating providers. Effective strategies to increase access to HCV care in rural areas are needed. We evaluated a model of telemedicine-based HCV care.

**Figure:**
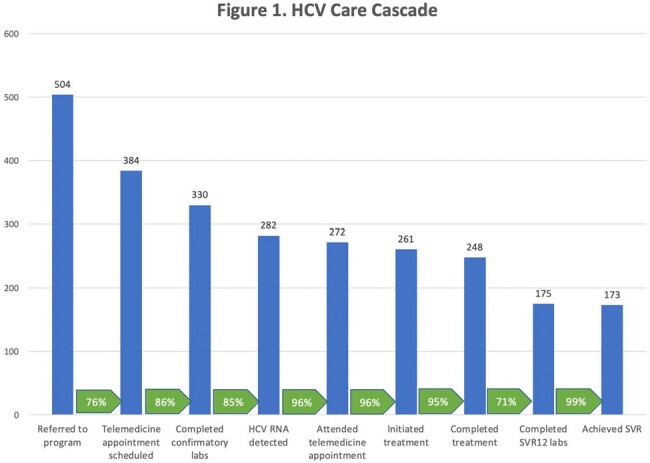

Note: At time of analysis 4 patients were currently on treatment and 9 were not yet due for SVR-12

**Methods:**

The TRAnsporting Hep C **V**iral **EL**imination Services via Telemedicine (TRAVEL) program was implemented by the Johns Hopkins Viral Hepatitis Center in partnership with the Allegany County Health Department in rural Maryland. The program provides low-barrier access to HCV care via a blended model of telemedicine with high touch in-person nurse case management locally and specialty pharmacy support and clinicians remotely. Appointment, treatment, SUD diagnoses and laboratory information were captured from the electronic medical record. Treatment initiation and completion were confirmed by pharmacy records. Sustained virologic response (SVR) was defined as HCV RNA < 15 IU/ml measured >12 weeks after the end of treatment. Outcome by SUD diagnosis was compared using descriptive statistics (chi squared) and significance set at p < 0.05.

**Figure:**
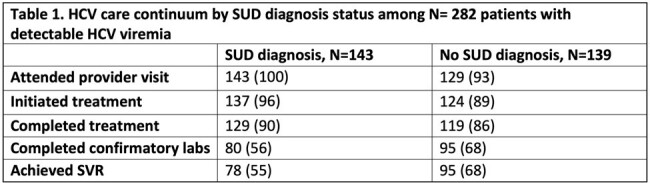

**Results:**

Between January 2018 and March 2024, 504 patients were referred to the program of which 384 (76%) scheduled an appointment. Scheduled patients were predominantly white (346, 90%) and male (228, 59%), with a median age of 42 years (Interquartile range (IQR) 36,55). Overall, (181, 47%) had a SUD, including opioid (162, 42%), cocaine (13, 3%), methamphetamine (6, 2%), and alcohol (33, 9%). 330 (86%) completed HCV RNA testing and 282 (73%) had HCV viremia. Of patients with HCV viremia, 272 (96%) attended a provider visit, 261 (93%) initiated treatment, 248 (88%) completed treatment, 175 (62%) completed SVR labs and 173 (61%) had confirmed SVR. Among those with SVR testing, 173/175 (99%) achieved SVR, and SVR in those with and without SUD was 98% (78/80) and 100% (95/95) respectively, p-value = 0.40.

**Conclusion:**

In a rural community with a high prevalence of SUDs and HCV, a health department-based telemedicine program offering intensive, local nurse case management and remote expert clinician and pharmacist care achieved high retention in care and HCV cure rates. While most patients who completed treatment achieved HCV cure, strategies are needed to increase laboratory assessments of response.

**Disclosures:**

Sherilyn Brinkley, MSN, CRNP, AbbVie: Honoraria|Gilead Sciences: Honoraria Mark S. Sulkowski, MD, AbbVie: Advisor/Consultant|Aligos Therapeutics: Advisor/Consultant|Gilead: Advisor/Consultant|GSK: Advisor/Consultant|GSK: Grant/Research Support|Janssen: Grant/Research Support|Precision Biosciences: Advisor/Consultant|Vir: Grant/Research Support|Virion: Advisor/Consultant Oluwaseun Falade-Nwulia, MBBS ,MPH, Abbvie Inc: Grant/Research Support|Gilead Sciences: Advisor/Consultant

